# Transcriptomic analysis identifies novel potential biomarkers and highlights cilium-related biological processes in the early stages of prion disease in mice

**DOI:** 10.1080/19336896.2022.2095186

**Published:** 2022-07-03

**Authors:** Yong-Chan Kim, Byung-Hoon Jeong

**Affiliations:** a Korea Zoonosis Research Institute, Jeonbuk National University, Iksan, Republic of Korea; b Department of Bioactive Material Sciences and Institute for Molecular Biology and Genetics, Jeonbuk National University, Jeonju, Republic of Korea

**Keywords:** Prion, scrapie, transcriptome, RNA sequencing, cilium, bioinformatics, hippocampus

## Abstract

Prion diseases are fatal and irreversible neurodegenerative diseases induced by the pathogenic form of the prion protein (PrP^Sc^), which is converted from the benign form of the prion protein (PrP^C^). These diseases are characterized by an extended asymptomatic incubation period accompanied by continuous conversion of PrP^C^ to PrP^Sc^. However, to date, the mechanism governing the conversion to PrP^Sc^ in the initial stages of prion disease has not been fully elucidated. We collected transcriptome data from the hippocampus of wild-type mice and prion-infected mice at 8 weeks post injection from the Gene Expression Omnibus and analysed differentially expressed genes and related signalling biological process using bioinformatic tools. We identified a total of 36 differentially expressed genes, including 22 upregulated genes and 14 downregulated genes. In addition, we identified that the cilium-related biological process was enriched in the early stages of prion disease. Furthermore, up- and down-regulated genes were associated with cilium-related cellular components and synapse-related cellular components, respectively. To the best of our knowledge, our study was the first to observe the upregulation of cilium-related genes in the early stages of prion disease.

## Introduction

Prion diseases are fatal and chronic neurodegenerative diseases caused by a deleterious form of the prion protein (PrP^Sc^) that is converted from the normal form of the prion protein (PrP^C^) [1–8]. These diseases are characterized by an extended asymptomatic incubation period accompanied by the continuous conversion of PrP^C^ to PrP^Sc^ and the exponential manifestation of disease progression, including ataxia, hallucinations, muscle stiffness and dementia in the terminal stage [9,10]. To date, since drugs that can be employed to treat patients after diagnosis have not been developed for prion diseases, they are regarded as incurable and irreversible [11–13]. Thus, several studies have been conducted to identify key factors that may be involved in the conversion process of PrP^C^ to PrP^Sc^.

Previous studies have reported that linear polyanions, including glycosaminoglycans (GAG), and heparin sulphate, enhance the conversion efficiency in models of prion disease [14,15]. In addition, host-encoded RNAs, including synthetic RNA and RNA from the liver, were also associated with the conversion rate of PrP^C^ to PrP^Sc^ [16,17]. Furthermore, phosphatidylethanolamine, a major phospholipid in the membranes of eukaryotic cells, plays an important role in the generation of infectious prions [18]. However, these fragmentary studies cannot explain cellular processes, which are primarily involved in the conversion process of PrP^C^ to PrP^Sc^. Recently, in two studies using RNA sequencing (RNA-Seq), prion-infected mice commonly displayed enhanced glial-related signatures in astrocytes and microglia accompanied by clinical signs of prion diseases [19,20]. However, these studies primarily investigated prion-infected mice 80 days post injection and used intracranial inoculated mice, which showed rapid disease progression. Thus, these studies cannot provide a view of the cellular process in the initial stages of the conversion of PrP^C^ into PrP^Sc^.

Thus, in the present study, to identify PrP^Sc^ conversion-related cellular processes in the initial stages of prion disease, we performed transcriptomic analyses at the early stage of prion disease and identified potential novel biomarkers and related biological processes. In addition, we collected the original transcriptomic dataset in the hippocampus of intraperitoneal injected wild-type mice and prion-infected mice at 8 weeks post injection, from the Gene Expression Omnibus and analysed differentially expressed genes and related biological processes using bioinformatic tools.

## Results

### Identification of potential novel biomarkers at the early stage of prion disease in mice

**Figure 1.**
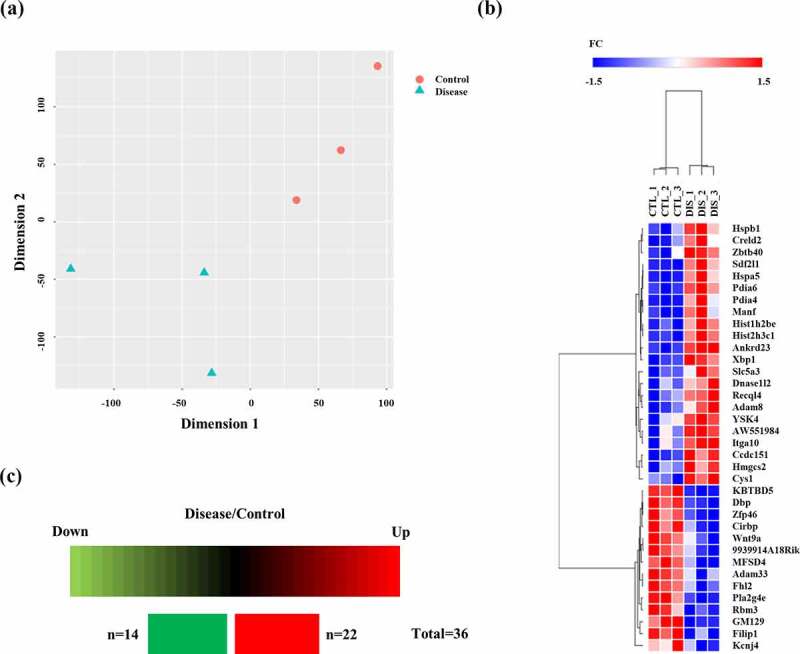
(a) t-distributed stochastic neighbour embedding (t-SNE) plot visualizing the separation of transcriptomes in the hippocampus of wild-type mice (n = 3) and RML scrapie-infected mice (n = 3) at 8 weeks post injection. (b) The heatmap was visualized for fold change of all differentially expressed genes in the hippocampus of prion-infected mice at 8 weeks post injection, showing the level of relative increase (red) and decrease (blue). Hierarchical clustering on the differentially expressed genes was performed based on one minus Pearson correlation using average linkage method. (c) Summary of differentially expressed genes in the prion-infected mice at 8 weeks post injection. The number of differentially expressed genes showing the level of relative increase and decrease.

We analysed the transcriptomes in the hippocampus of wild-type mice and prion-infected mice at 8 weeks post injection. Detailed information on the dataset analysed in the present study is presented in Table 1. Total transcriptomic datasets without threshold restrictions were used to perform t-distributed stochastic neighbour embedding (t-SNE) analysis to compare the transcriptome data response to prion infection at 8 weeks post injection. The t-SNE plot showed the separated clusters of each transcriptome dataset for prion-infected mice and wild-type mice (Figure 1(a)).

**Table 1.** Detailed information on the dataset analysed in this study.

Characteristics	
Target organ	Brain, hippocampus
Methods	RNA Sequencing
Prion disease agent	Scrapie, Rocky Mountain Laboratory (RML) strain
Inoculation route	Intraperitoneal injection
Time point of sacrifice	8 weeks postinjection
Number of mice	Wild-type mice, n = 3
	Prion-infected mice, n = 3

Potential biomarkers in the hippocampus of prion-infected mice at the early stage of prion disease were further extracted based on multivariate statistical analysis according to the conditions with the parameters of differentially expressed genes satisfied with |FC>1.5| and FDR<0.05. The differentially expressed genes at 8 weeks post injection were visualized by a heatmap. In addition, hierarchical clustering was performed on the differentially expressed genes. The dendrogram of clustering analysis showed that the differentially expressed genes could completely separate prion-infected mice from wild-type mice (Figure 1(b)). Furthermore, a total of 36 genes showed alterations at 8 weeks post injection in prion-infected mice. In the mouse brain at 8 weeks post injection, 22 genes and 14 genes showed upregulation and downregulation, respectively (Figure 1(c)). Detailed information on the differentially expressed genes is presented in Table 2. The most upregulated gene was the stromal cell derived factor 2 like 1 (*Sdf2l1*) gene followed by the heat shock protein family B (small) member 1 (*Hspb1*) gene and histone H2B type 1-C/E/F/G/I (*Hist1h2be*) gene. The most downregulated gene was the ADAM metallopeptidase domain 33 (*Adam33*) gene followed by the phospholipase A2 Group IVE (*Pla2g4e*) gene and Wnt family member 9A (*Wnt9a*) gene (Table 2).

**Table 2.** List of differentially expressed genes between wild-type and prion-infected mice at 8 weeks post injection.

Gene	Regulation	Log_2_(Fold Change)	*P*-value
*Sdf2l1*	Up	1.11805	2.80E-05
*Hspb1*	Up	1.093554	1.61E-02
*Hist1h2be*	Up	1.050371	4.96E-03
*Hspa5*	Up	0.984737	8.17E-12
*YSK4*	Up	0.924134	3.63E-02
*Pdia4*	Up	0.913124	4.89E-06
*Hist2h3c1*	Up	0.896597	6.60E-03
*Slc5a3*	Up	0.891149	6.86E-05
*Dnase1l2*	Up	0.884045	2.84E-02
*AW551984*	Up	0.882569	4.56E-02
*Recql4*	Up	0.881429	1.27E-02
*Itga10*	Up	0.809414	6.08E-03
*Adam8*	Up	0.791209	2.11E-03
*Creld2*	Up	0.788257	7.02E-03
*Ankrd23*	Up	0.758375	4.97E-02
*Pdia6*	Up	0.743047	2.18E-11
*Manf*	Up	0.739741	3.04E-04
*Ccdc151*	Up	0.736193	3.83E-02
*Hmgcs2*	Up	0.691802	2.85E-02
*Zbtb40*	Up	0.680225	6.05E-03
*Cys1*	Up	0.660823	5.25E-03
*Xbp1*	Up	0.660348	4.18E-09
*Adam33*	Down	−1.97937	2.14E-03
*Pla2g4e*	Down	−1.61341	2.95E-12
*Wnt9a*	Down	−1.49813	8.64E-06
*KBTBD5*	Down	−1.17323	1.47E-03
*Dbp*	Down	−1.10466	8.49E-15
*GM129*	Down	−1.01206	2.93E-06
*9930014A18Rik*	Down	−0.96725	9.16E-03
*Zfp46*	Down	−0.93888	4.28E-12
*Filip1*	Down	−0.85188	1.71E-04
*Cirbp*	Down	−0.77537	2.85E-06
*MFSD4*	Down	−0.71421	6.68E-07
*Kcnj4*	Down	−0.70145	5.25E-03
*Fhl2*	Down	−0.62938	3.48E-04
*Rbm3*	Down	−0.59719	3.35E-05

### Biological process analysis at the early stage of prion disease in mice

**Figure 2.**
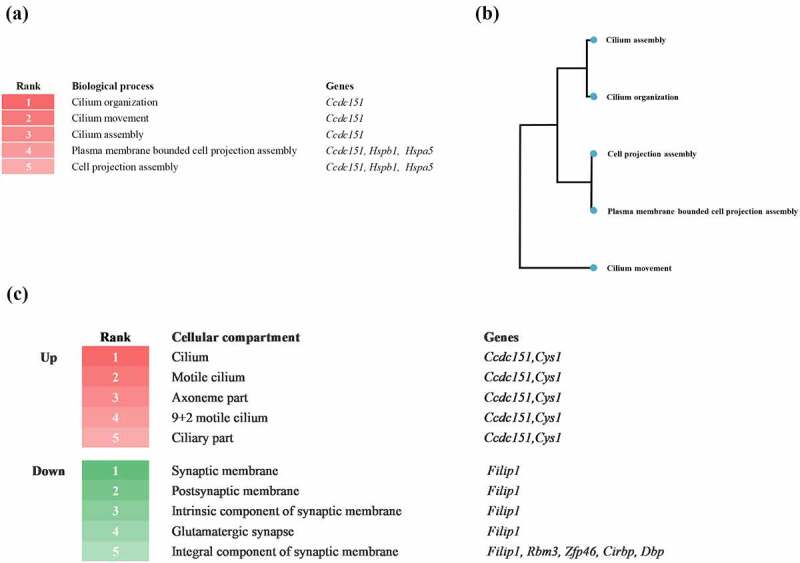
(a) Heatmap displaying the top five signalling biological processes enriched at 8 weeks post injection. Numbers and colours indicate the ranking of the respective biological processes. (b) Biological process tree displaying the relationship of the top five signalling biological processes enriched at 8 weeks post injection. (c) Heatmap displaying the top five upregulated (red) and downregulated (green) cellular compartments at 8 weeks post injection. Numbers and colours indicate the ranking of the respective cellular compartments.

To investigate the differentially expressed genes at the early stage of the prion disease-related biological process, we performed biological process analysis by GAGE based on a database source, gene ontology (GO) biological process and GO cellular component (Figure 2). The heatmap shows the top five biological processes enriched at 8 weeks post injection. Notably, prion-infected mice at the early stage showed enhancement of cilia and cell projections. The three biological processes, including cilium organization, movement, and assembly, were associated with the coiled-coil domain containing 151 (*Ccdc151*) gene. The two biological processes, including plasma membrane bounded cell projection assembly and cell projection assembly, were related to *Ccdc151*, *Hspb1*, and heat shock protein family A member 5 (*Hspa5)* genes (Figure 2(a)). The biological process tree was used to visualize the clusters of the biological processes (Figure 2(b)). Next, we analysed differentially expressed gene-related cellular components (Figure 2(c)). The upper heatmap depicts the top five upregulated cellular components at 8 weeks post injection. Notably, prion-infected mice at the early stage showed upregulation in the cilium, motile cilium, axoneme part, 9 + 2 motile cilium and ciliary part. The five cellular compartments were commonly related to *Ccdc151* and cystin 1 *(Cys1)* genes. The lower heatmap depicts the top five downregulated cellular components at 8 weeks post injection. Notably, prion-infected mice at the early stage showed downregulation of the synaptic membrane, postsynaptic membrane, intrinsic component of synaptic membrane, glutamatergic synapse and integral component of synaptic membrane. The four cellular compartments, including synaptic membrane, postsynaptic membrane, intrinsic component of synaptic membrane, and glutamatergic synapse were associated with filamin A interacting protein 1 (*Filip1*) gene. The one cellular compartment, including integral component of synaptic membrane, was related to *Filip1*, RNA-binding protein 3 (*Rbm3*), Zinc finger protein 46 (*Zfp46*), cold-inducible RNA-binding protein (*Cirbp*) and D-Box binding PAR BZIP transcription factor (*DBP*) genes.

## Discussion

In the present study, we first reported that cilium-related genes are significantly differentially expressed in the hippocampus of prion-infected mice in the early stages of prion disease. In the previous study, the authors investigated primarily intracerebral-inoculated prion-infected mice and found 813 differentially expressed genes in the hippocampus of prion-infected mice at 8 weeks post injection [19]. However, since the disease progression was fairly rapid in intracerebral inoculated prion-infected mice, it is difficult to observe the cellular mechanism in the initial state of prion disease. Thus, we obtained information of the intraperitoneal inoculated prion-infected mice from the original dataset and analysed it to investigate the cellular process in the initial stages of prion diseases. Since the intraperitoneal inoculated prion-infected mice showed a relatively long incubation period compared to the intracerebral prion-infected mice and a previous study has reported that detectable PrP^Sc^ was found in intraperitoneally infected rodent brain at 2 days post inoculation, we have assumed that it is suitable to investigate the physiological early stage of prion diseases in the mice at 8 weeks post injection [21]. In the present study, we identified a total of 36 differentially expressed genes in the hippocampus of prion-infected mice at 8 weeks post injection. Although the strongly altered genes, including *Sdf2l, Hspb1* and *Hist1h2be* genes were not involved in cilium-related biological processes, a cilium-related gene, *Ccdc151* gene was most frequently involved in the cilium-related biological processes according to the bioinformatics analysis (Figure 2(a)). In addition, the cilium-related biological process was regulated by a relatively small protein set, including tetratricopeptide repeat protein 25 (Ttc25), protein tilB homolog (Lrrc6), transcription factor RFX3 (Rfx3), inversin (Invs), oral-facial-digital syndrome 1 protein homolog (Ofd1) dynein, axonemal, heavy chain 11 (Dnah11), Ccdc39, 40, and 151 (data not shown). Among these proteins, the Ccdc151 protein, which is encoded by *Ccdc151* gene, was expressed in primary and motile cilium and is the crucial factor for cilium function. Previous studies have reported that non-synonymous mutations in Ccdc151 protein are associated with primary ciliary dyskinesia via disruption of the formation of the outer dynein arm docking complex [22]. In addition, a nonsense mutation in Ccdc151 protein also caused primary ciliary dyskinesia [23]. Furthermore, the Ccdc151 protein plays a pivotal role in IFT-dependent motile cilium [24]. However, it is elusive whether the alteration of the *Ccdc151* gene is specific in the early stage of prion disease. Thus, further investigation of Ccdc151 protein at all stages of prion disease is highly desirable.

In the present study, we observed altered cilium-related biological processes in intraperitoneally prion-infected mice but not in intracerebrally prion-infected mice. These results indicate the possibility that cilium-related changes are not universal early alterations in gene expression in response to prion infection. However, since the disease progression after an intracerebral infection is relatively faster than that after an intraperitoneal infection, the biological process identified in an extremely early stage of intraperitoneally prion-infected mice may have not been observed in intracerebrally prion-infected mice. Additionally, since the infection route in an intraperitoneal prion infection differs from that in an intracerebral prion infection, unique altered cilium-related molecules may have been found. A further comparative study targeting cilium-related molecules in intraperitoneally and intracerebrally prion-infected mice is needed to find the difference between these two infection types.

The cilium is an organelle projected from the cell body and classified into 2 types: nonmotile and motile cilia [25]. Among these cilia, the nonmotile cilium, also called the primary cilium, is expressed in almost every type of cell and acts as a master regulator of cellular signalling [26,27]. Conversely, the motile cilium is generally expressed in the genital organs or airways and involved in movement and clearance. However, in the brain, motile cilia are primarily expressed in ependymal cells and are involved in the brain waste clearing system via ventricles [28–30]. Recently, the alteration of brain waste clearing system-related proteins was observed in prion diseases and activation of cerebrospinal fluid (CSF) circulation showed dramatic clearance of PrP^Sc^ in brain lesions [7]. Thus, the alteration of the cilium-related genes may be induced by upregulation of clearance. In addition, a previous study has reported that prion-induced photoreceptor degeneration begins with PrP^Sc^ accumulation in cones at two distinct sites including cilia and ribbon synapses. This result indicated the association of cilium-related biological processes with prion disease in the retina [31]. Although there was no validation at the RNA level for the differentially expressed genes found in this study, since several pieces of evidence have indicated the association of cilium with prion diseases, further investigation of cilium in prion diseases at the protein level is highly desirable in the future. Specifically, investigation of the up- and down-regulated genes at the protein level in body fluids of Creutzfeldt-Jakob disease (CJD) patients and prion-infected mice is highly beneficial to validate the possibility of biomarkers.

Notably, since differentially expressed genes at the early stage of prion diseases are significantly associated with the cilium, we can postulate that the cilium plays an essential role in the early stage of prion disease when considering innate physiological functions of the cilium. In future research, further validation through the analysis of prion disease in mice for which cilium-related gene, *Ccdc151* gene are knocked out is warranted to find a novel biomarker.

In addition, a recent study reported that PrP^C^ is located on the primary cilium in stem and progenitor cells from the central nervous system and plays pivotal roles in cilium-dependent signalling, including sonic hedgehog signalling and α-tubulin posttranslational modifications in neuroepithelial cells [32]. Furthermore, PrP^C^ participates in the self-renewal function of neural stem cells via the Notch signalling pathway [33,34]. Since PrP^C^ is associated with the self-renewal function and is located on the cilium, using single-cell RNA-Seq to further investigate the relationship between PrP^C^ and the cilium in prion disease is warranted.

Furthermore, we observed that upregulated genes were related to cilium-related cellular components; however, downregulated genes were associated with synapse-related cellular components. In a previous study, mice infected with prions via the intrahippocampal route displayed a pronounced decrease in the number of synapses in the striatum and showed impairment of the presynaptic compartment and loss of dendritic spines in CA1 pyramidal neurons [35]. In addition, CJD patients also showed substantial loss of neurons accompanied by synaptic degeneration. Our findings indicate that synapse-related alterations are already underway in the early stages of prion disease without clear PrP^Sc^ deposition [36]. Further study of the association between the downregulated genes and synapse function in prion disease is merited.

In conclusion, we compared the transcriptomes in the hippocampus of wild-type and prion-infected mice at 8 weeks post injection and identified a total of 36 differentially expressed genes. We determined that the cilium-related signalling biological processes were enhanced in the early stages of prion disease. In addition, upregulated and downregulated genes were related to cilium-related cellular components and synapse-related cellular components, respectively. To the best of our knowledge, this report describes the first bioinformatic analysis on the association between prion disease and cilia in the early stages of prion disease.

## Methods

### Data collection

The raw dataset of the transcriptome in the hippocampus of intraperitoneal inoculated wild-type and prion-infected mice at 8 weeks post injection analysed in the present study were obtained from Gene Expression Omnibus (accession number: GSE144738).

### Data analysis

The raw data was extracted as read counts format. Differentially expressed genes were identified based on FC>1.5 and FDR<0.05 with statistical significance (P < 0.05) using the R package edgeR from Bioconductor (version 3.0). Hierarchical clustering on the differentially expressed genes was performed using the GENE-E program (Broad Institute, MA, USA). The distances between genes were calculated based on one minus Pearson correlation using the average linkage method. The t-SNE method was used for projecting high-dimensional gene expression data in 2D space using Python. biological process enrichment analysis was performed using the R package GAGE form Bioconductor (version 3.0) based on database sources, including GO biological processes and GO cellular components (http://geneontology.org) based on FDR<0.1.
